# Worldwide AI ethics: A review of 200 guidelines and recommendations for AI governance

**DOI:** 10.1016/j.patter.2023.100857

**Published:** 2023-10-13

**Authors:** Nicholas Kluge Corrêa, Camila Galvão, James William Santos, Carolina Del Pino, Edson Pontes Pinto, Camila Barbosa, Diogo Massmann, Rodrigo Mambrini, Luiza Galvão, Edmund Terem, Nythamar de Oliveira

**Affiliations:** 1 Graduate Program in Philosophy, Pontifical Catholic University of Rio Grande do Sul, Porto Alegre, Rio Grande do Sul, Brazil; 2 Graduate Program in Philosophy, University of Bonn, Bonn, North Rhine-Westphalia, Germany; 3 Graduate Program in Law, Pontifical Catholic University of Rio Grande do Sul, Porto Alegre, Rio Grande do Sul, Brazil; 4 Psychology Undergraduate Program, Pontifical Catholic University of Rio Grande do Sul, Porto Alegre, Rio Grande do Sul, Brazil; 5 Law Undergraduate Program, Pontifical Catholic University of Rio Grande do Sul, Porto Alegre, Rio Grande do Sul, Brazil; 6 Graduate Program in Philosophy, University of Johannesburg, Porto Alegre, Johannesburg, South Africa

**Keywords:** artificial intelligence, machine learning, ethics, ethics of artificial intelligence, guidelines

## Abstract

The utilization of artificial intelligence (AI) applications has experienced tremendous growth in recent years, bringing forth numerous benefits and conveniences. However, this expansion has also provoked ethical concerns, such as privacy breaches, algorithmic discrimination, security and reliability issues, transparency, and other unintended consequences. To determine whether a global consensus exists regarding the ethical principles that should govern AI applications and to contribute to the formation of future regulations, this paper conducts a meta-analysis of 200 governance policies and ethical guidelines for AI usage published by public bodies, academic institutions, private companies, and civil society organizations worldwide. We identified at least 17 resonating principles prevalent in the policies and guidelines of our dataset, released as an open source database and tool. We present the limitations of performing a global-scale analysis study paired with a critical analysis of our findings, presenting areas of consensus that should be incorporated into future regulatory efforts.

## Highlights

• Significant growth in AI research and industry has led to an “AI ethics boom”
• Much of the normative discourse in AI ethics comes as guidelines
• A thorough description of these guidelines remains lacking
• We present a descriptive analysis of 200 AI guidelines in an open source fashion

## The bigger picture

After the AI winter in the late 80s, AI research has experienced remarkable growth. Currently, a lot of work is taking place to define the values and ideas that should guide AI advances. A key challenge, however, lies in establishing a consensus on these values, given the diverse perspectives of various stakeholders worldwide and the abstraction of normative discourse. Researchers and policy makers need better tools to catalog and compare AI governance documents from around the world and to identify points of divergence and commonality.

## Teaser

Given the rising hype surrounding AI, much attention has been given to the question “what values should guide this development?” From moratoriums to the AI industry to endless lists of principles, it is easy to get lost in this normative discourse. Hence, to help determine whether a global consensus exists regarding our values concerning AI, this paper conducts a meta-analysis of 200 governance policies and guidelines for AI usage published by different stakeholders worldwide.

## Introduction

Since the last period of reduced interest in artificial intelligence (AI), the "AI winter" from 1987 to 1993, the field of AI research and industry has witnessed significant growth. This growth encompasses various aspects, including the development of new technologies, increased investment, greater media attention, and expanded capabilities of autonomous systems. A study analyzing the submission history on ArXiv from 2009 to 2021 reveals that computer science-related articles have become the most prevalent type of material submitted, increasing 10-fold starting in 2018. Furthermore, within the broad scope of computer science, the most frequently submitted sub-categories for publications are "computer vision and pattern recognition," "machine learning," and "computation and language,"^1^ i.e., areas where machine learning (a sub-field of AI research) has established itself as the reigning paradigm.

Moreover, investment in AI-related companies and startups has reached unprecedented levels, with governments and venture capital firms investing over $90 billion (USD) in the United States alone in 2021, accompanied by a surge in the registration of AI-related patents.^2^ While these money-field advancements have brought numerous benefits, they also introduce risks and side effects that have promoted several ethical concerns, like risks to user privacy, the potential for increased surveillance, the environmental cost of the industry, and the amplification of prejudices and discrimination on a large scale, which can disproportionately harm vulnerable groups. Consequently, the expansion of the AI industry has given rise to what we refer to as the "AI ethics boom," i.e., a period marked by an unprecedented demand for regulation and normative guidance in this field.

One of the central questions surrounding this boom is the determination of what ethical premises should guide the development of AI technologies. And to answer this question, a plethora of principles and guidelines have been proposed by many stakeholders. However, the consensus and divergences in these varied discourses have yet to be extensively accessed. For instance, do Silicon Valley-based companies follow the same precautions as major Chinese technology firms? Are these concerns relevant to end-users in countries with diverse cultural and social norms? Establishing a consensus to support the global regulations currently under discussion is of paramount importance in both a practical and theoretical sense.

To address these questions, we draw inspiration from previous works by meta-analysts and meticulously survey a wide array of available ethical guidelines related to AI development. These sources include governance policies of private companies, academic institutions, and governmental and non-governmental organizations, as well as ethical guidelines for AI usage. By analyzing 200 documents in five different languages, we gathered information on what ethical principles are the most popular, how they are described, where they come from, their intrinsic characteristics, and much else. Our primary goal was to identify the most advocated ethical principles, to examine their global distribution, and to assess if there is a consistent understanding of these principles. Ultimately, this analysis aims to determine whether a consensus exists regarding the normative discourse presented in ethical guidelines surrounding AI development.

### State of the art

One of the first studies to promote a meta-analysis of published AI ethical guidelines was that of Jobin et al.^3^ In this study, these authors sought to investigate whether a global agreement on emerging questions related to AI ethics and governance would arise. The research identified 84 documents containing ethical guidelines for intelligent autonomous systems using the Preferred Reporting Items for Systematic Reviews and Meta-Analyses framework (originally developed for analysis in the healthcare sector).^4^ At the time, some of them were one of the most cited guidelines in the literature, like the Organization for Economic Co-operation and Development (OECD) Recommendation of the Council on Artificial Intelligence,^5^ the High-Level Expert Group on AI Ethics Guidelines for Trustworthy AI,^6^ the University of Montréal Declaration for responsible development of artificial intelligence,^7^ the Villani Mission’s French National Strategy for AI,^8^ among many others.

Jobin et al.’s^3^ sample also contained documents from governmental organizations (e.g., Australian Government Department of Industry Innovation and Science^9^), private companies (e.g., SAP,^10^ Telefonica,^11^ IBM^12^), non-governmental organizations (e.g., Future Advocacy,^13^ AI4People^14^), non-profit organizations (e.g., Internet Society,^15^ Future of Life Institute^16^), academic institutions (e.g., AI Now Institute^17^), and professional associations (e.g., IEEE^18^), among other types of institutions, representing a multi-stakeholder sample that for years was the most extensive collection of AI guidelines analyzed systematically.

One of the main findings in Jobin et al.’s^3^ work was the detection of the most common ethical principles in the discourse of the evaluated documents, those being transparency, justice/equity, non-maleficence, accountability, privacy, beneficence, freedom and autonomy, trust, dignity, sustainability, and solidarity. Of these 11 ethical principles cited, five were the most recurrent: transparency (86%), justice (81%), non-maleficence (71%), responsibility (71%), and privacy (56%). Furthermore, Jobin et al.’s work is careful not to impose any normative guidelines for the effectiveness of the mentioned principles. It attempts to raise the issue and map the global picture in a pioneer work of descriptive ethics. However, their limited sample, where, for example, no Latin American countries are mentioned, makes the true global representativeness of the results questionable, a limitation recognized by Jobin et al. as one of their blind spots.

Thilo Hagendorff^19^ conducted another study that presented a similar type of analysis. In his research, Hagendorff focused on a smaller sample of 21 documents. He excluded those older than 5 years and that only addressed a national context unrelated to AI, such as data science and robotics. Furthermore, Hagendorff did not consider corporate policies but deliberately selected documents deemed relevant in the international discourse (IEEE, Google, Microsoft, and IBM) based on his evaluation criteria. Even working with a smaller sample, Hagendorff’s findings corroborate with those of Jobin et al.,^3^ where the most mentioned principles found were accountability (77%), privacy (77%), justice (77%), and transparency (68%). Hagendorff (like Jobin et al.) also mentions the underrepresentation of institutions in South America, Africa, and the Middle East as a clear bias of his sample.

Hagendorff is also more critical in his analysis, presenting concluding points such as the following:

• The lack of attention given to questions related to labor rights, technological unemployment, the militarization of AI, the spread of disinformation, and the misuse/dual-use of AI technology
• The lack of gender diversity in the tech industry and AI ethics
• The short, brief, and minimalist views some documents give to normative principles
• The lack of technical implementations for how to implement the defended principles in AI development
• The lack of discussion on long-term risks (e.g., artificial general intelligence safety, existential risks)

While the works of Hagendorf and Jobin et al. are valuable contributions to AI ethics, we question the criteria for filtering documents used by these works. We argue that if we want to investigate the consensus regarding the normative dispositions of different countries and organizations regarding AI, we should not use popularity-based filtering. In other words, a descriptive ethics evaluation should take as many viewpoints as possible if it aims to make a solid description of the "global landscape."

As a last mention, we would like to cite the work done by Fjeld et al.,^20^ one of the first to present documents from Latin America. In their study, Fjeld et al. worked with 36 samples produced by a variety of institution types, but as with Hagendorff,^19^ Fjeld et al. also excluded data science, robotics, and other AI-related fields/applications. According to these authors, eight principles were the most commonly cited in their sample: fairness/non-discrimination (present in 100% of the analyzed documents), privacy (97%), accountability (97%), transparency/explainability (94%), safety/security (81%), professional responsibility (78%), human control of technology (69%), and promotion of human values (69%).

Like in the study of Jobin et al.,^3^ Fjeld et al.^20^ cite the variability in how such principles are defined as an important element to be addressed. For example, in the 2018 version of the Chinese Artificial Intelligence Standardization White Paper,^21^ the authors mention that AI can serve to obtain more information from the population, even beyond the data that has been consented to (i.e., violation of informed consent would not undermine the principle of privacy), while the Indian National Strategy for Artificial Intelligence Discussion Paper (National Institution for Transforming India)^22^ argues that its population must become massively aware so that they can effectively consent to the collection of personal information. We consider the delineation of diverging principles across documents to be one of the standout strengths of Fjeld et al.’s work, which warrants replication on a broader scale.

There is more meta-analytical work that has been done in AI ethics that we will not cite in depth. For a complete review of meta-analytical research on normative AI documents, we recommend the work done by Schiff et al.,^23^ which cites many other important works.

Now, shifting the perspective from past works to our own, we argue that many of the mentioned analyses, following Jobin et al.’s^3^ work, suffer from a small sample size. While works like the ones done by Hagendorff^19^ and Fjeld et al.^20^ show more diversified categories of typologies or sample sizes, their limited sample may hinder the generalization of their results. Meanwhile, Jobin et al. do not present some features (e.g., an extensive exemplification of how principles diverge) presented in other works that used a smaller sample. Also, we would like to point out that none of these studies released their dataset in a form that would allow the replication of their findings, which makes all of these studies (if one would not be willing to redo all the work) irreproducible.

To cover these shortcomings, we propose an updated review of AI guidelines and related literature: *Worldwide AI Ethics*.

### Methodology

From the gaps pointed out in the previous meta-analyses, in this study, we sought to present the following to the AI community.

(1) A quantitatively larger and more diverse sample size, as done by Jobin et al.^3^ Our sample possesses 200 documents originating from 37 countries, spread over six continents, in five different languages.
(2) Combined with a more granular typology of document types, as done by Hagendorff.^19^ This typology allowed an analysis beyond the mere quantitative regarding the content of these documents.
(3) Presented in an insightful data visualization framework. We believe the data presentation done by authors like Hagendorff and Fjeld et al.^20^ was not "user-friendly" or clear, something that we tried to overcome in our work.
(4) Released with an open source dataset, making our work reproducible and extendable.

The focus of this study is guidelines related to the ethical use of AI technologies. We refer to "guidelines" as documents conceptualized as recommendations, policy frameworks, legal landmarks, codes of conduct, practical guides, tools, or AI principles for the use and development of this type of technology. The resonating foundation for most of these documents is the presence of a form of “principlism,”^24^ i.e., the use of ethical principles to support normative claims.

Now, deconstructing the expression "AI technologies," with "AI," our scope of interest encompasses areas that inhabit the multidisciplinary umbrella that is artificial intelligence research, such as statistical learning, data science, machine learning (ML), logic programming/symbolic AI, optimization theory, robotics, software development/engineering, etc. And with the term "technologies," we refer to specific tools/techniques, applications, and services. Thus, the term refers to technologies for automating decision processes and mimicking intelligent/expert behavior. Unlike previous works,^19^^,^^20^ we included areas disregarded previously (software development, data science, robotics).

### Sources

We used as sources for our sample two public repositories, the “AI Ethics Guidelines Global Inventory,” from AlgorithmWatch, and the “Linking Artificial Intelligence Principles*”* (LAIP) guidelines. The AlgorithmWatch repository contained 167 documents, while the LAIP repository contained 90.

Initially, we checked for duplicate samples between both repositories. After disregarding them, we also scavenged for more documents through web search engines and web scraping, utilizing search strings such as "Artificial Intelligence Principles," "Artificial Intelligence Guidelines," "Artificial Intelligence Framework," "Artificial Intelligence Ethics," "Robotics Ethics," "Data Ethics," "Software Ethics," and "Artificial Intelligence Code of Conduct," among other related search strings. We limited our search to samples written/translated in one of the five languages our team could cope with: English, Portuguese, French, German, and Spanish.

We were able to collect in this manner 200 documents. Thus, after defining our pool of samples, we initiated the data collection stage. We divided this stage into two phases.

### First phase

In phase one, members of our team received different quotas of documents. Each team member was responsible for reading, translating when needed, and hand-coding pre-established features. The first features looked for were the following:

• Institution responsible for producing the document
• Country/world region of the institution
• Type of institution (e.g., academic, non-profit, governmental, etc.)
• Year of publication
• Principles (as done by Fjeld et al.,^20^ we broke principles into themes of resonating discourse)
• Principles description (i.e., the words used in a document to define/support a given principle)
• Gender distribution among authors (inferred through a first name automated analysis)
• Size of the document (i.e., word count)

For the gender inference part, after removing documents with unspecified authors, we performed a name-based gender analysis. Given the variety/diversity that names can possess, it was necessary to use automation to infer gender encodes (male/female). To make an accurate inference, we also extracted (in addition to each author’s name) the most likely nationality associated with each name. For this, we used (in addition to the country/origin of each document) an API service that predicts the most likely nationality associated with a given name. Finally, we used another API service to infer gender based on first name plus nationality. You can find the code for our implementation in this repository: https://github.com/Nkluge-correa/worldwide_AI-ethics.

Regarding how we defined principles, in the first phase, we established a list of principles so our team could focus their search. We based these on past works mentioned in the "related works" section: accessibility, accountability, auditability, beneficence/non-maleficence, dignity, diversity, freedom/autonomy, human-centeredness, inclusion, intellectual property, justice/equity, open source/fair competition, privacy, reliability, solidarity, sustainability, and transparency/explainability.

We also used this phase to determine categories/types assigned to each document in the second phase. These types were determined by the following:

(1) The nature/content of the document
(2) The type of regulation that the document proposes
(3) The normative strength of this regulation
(4) The impact scope that motivates the document

The first type relates to the nature/content of the document.

• Descriptive: descriptive documents take the effort of presenting factual definitions related to AI technologies. These definitions serve to contextualize "what we mean" when we talk about AI.
• Normative: normative documents present norms, ethical principles, recommendations, and imperative affirmations about what such technologies should be used/developed for.
• Practical: practical documents present development tools to implement ethical principles and norms, be they qualitative (e.g., self-assessment surveys) or quantitative (e.g., debiasing algorithms for ML models).

We defined these first three categories as mutually inclusive (documents may have all these features combined). The second type relates to the form of regulation that the document proposes.

• Government regulation: this category is designed to encompass documents made by governmental institutions to regulate the use and development of AI, strictly (legally binding horizontal regulations) or softly (legally non-binding guidelines).
• Self-regulation/voluntary self-commitment: this category is designed to encompass documents made by private organizations and other bodies that defend a form of self-regulation governed by the AI industry itself. It also encompasses voluntary self-commitment made by independent organizations (NGOs, professional associations, etc.).
• Recommendation: this category is designed to encompass documents that only suggest possible forms of governance and ethical principles that should guide organizations seeking to use, develop, or regulate AI technologies.

We defined these categories as mutually exclusive (the presence of a feature excludes the other). The third type of document classification pertains to the normative strength of the proposed regulation mechanism. In this regard, we established two distinct categories, drawing upon the definitions provided by the “Innovative and Trustworthy AI.”^25^

• Legally non-binding guidelines: these documents propose an approach that intertwines AI principles with recommended practices for companies and other entities (i.e., soft law solutions).
• Legally binding horizontal regulations: these documents propose an approach that focuses on regulating specific uses of AI through legally binding horizontal regulations, such as mandatory requirements and prohibitions.

We defined them as mutually inclusive. The final type relates to the perceived impact scope that motivates the evaluated document. With impact scope, we mean the dangers and negative prospects regarding the use of AI that inspired the type of normative propositions used. For this, three final categories were defined and also posed as mutually exclusive.

• Short-termism: we designed this category to encompass documents in which the scope of impact and preoccupation focus mainly on short-term problems, i.e., problems we are facing with current AI technologies (e.g., algorithmic discrimination, algorithmic opacity, privacy, legal accountability).
• Long-termism: we designed this category to encompass documents in which the scope of impact and preoccupation focus mainly on long-term problems, i.e., problems we may come to face with future AI systems. Since such technologies are not yet a reality, we can classify these risks as hypothetical or, at best, uncertain (e.g., sentient AI, misaligned AGI, super-intelligent AI, AI-related existential risks).
• Short-termism and long-termism: we designed this category to encompass documents in which the scope of impact is both short and long-term, i.e., they present a "mid-term" scope of preoccupation. These documents address issues related to the short-termism category while also pointing out the mid/long-term impacts of our current AI adoption (e.g., AI interfering in democratic processes, autonomous weapons, existential risks, environmental sustainability, labor displacement, and the need for updating our educational systems).

We proceeded to the second phase after all 200 documents received this first evaluation.

### Second phase

While in the first phase of our analysis, our team reviewed the entirety of our sample (each team member with their assigned quota), in phase two, a single team member reviewed all the work performed by the first phase. We concluded this approach would result in a standardized final sample. Thus, all post-processed documents passed the same criteria (and evaluator). In cases where uncertainties between classifications arose, we reached a consensus as a team.

We sought to establish our types and categories as objectively as possible, defining observable features that should indicate each created category/type, as presented above (e.g., the mention of existential risks or AGI timelines in documents classified as "long-termism"). However, we recognize that, in this type of work, even objective parameters are perceived and analyzed by subjective entities. Even if our final result possesses evaluation biases (some of our types may still be subject to interpretation and discussion), we consensually validated them as a team.

Also, while the first phase helped us explore the principles used by the literature, we refined and expanded this list in phase two. For example, we determined that similar principles could be aggregated under the same category by expanding their name since they upheld resonant values and ideas, e.g., diversity/inclusion/pluralism/accessibility.

We followed a simple heuristic for the creation of these aggregated principles.

(1) They appear to be cited a sufficient number of times (>10).
(2) They could not be integrated into another category without redefining their previous description.

**Figure 1:**
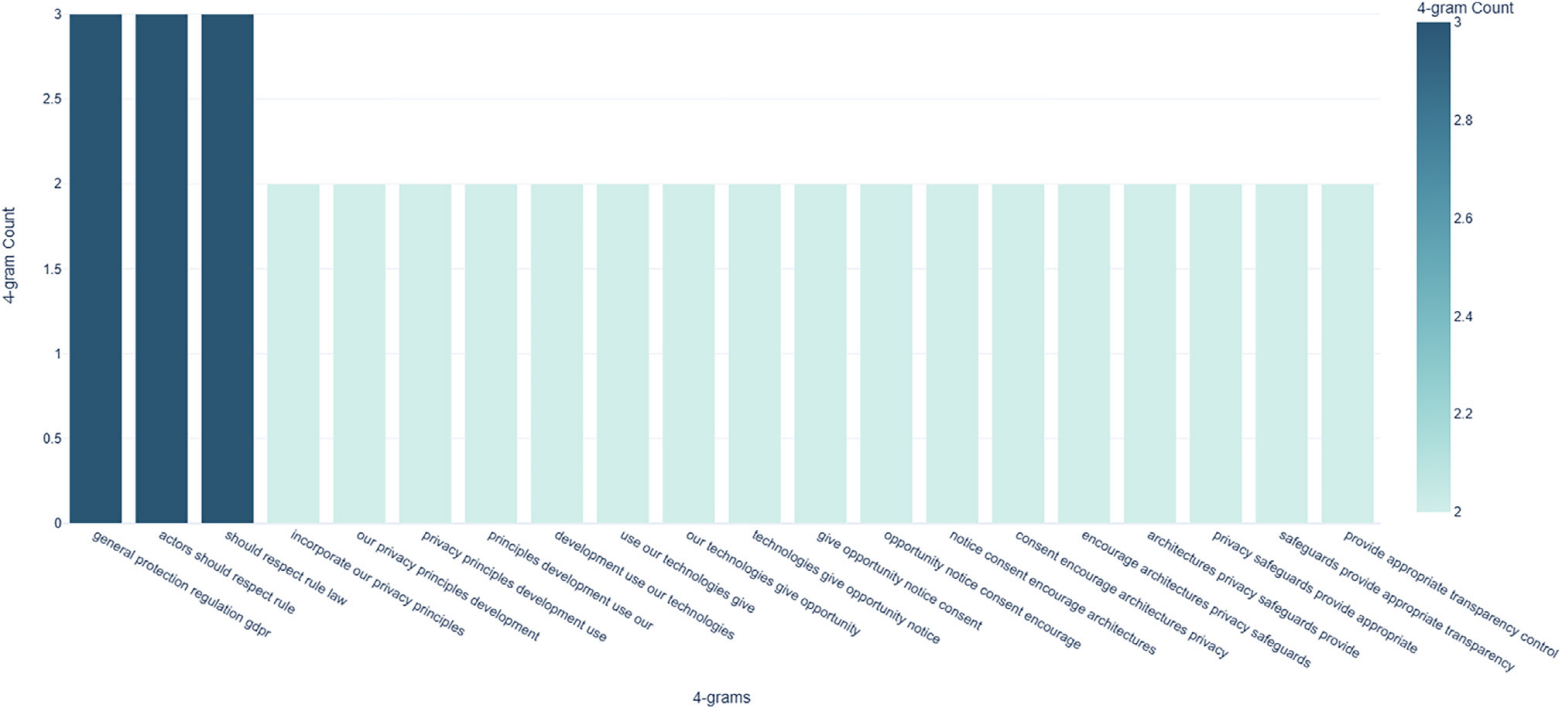
Top 20 recurrent 4-g for the 137 descriptions of "privacy"

We obtained the final description of these aggregated principles through a text mining technique known as n-gram analysis. This technique involved counting the successive repetition of words and groups of words within each principle category (Figure 1). The reader can find the code implementation of our approach in the following repository: https://github.com/Nkluge-correa/worldwide_AI-ethics.

The defined principles helped aggregate similar and resonating values while maintaining significant differences in word recurrence. Below, the reader can find the definition that we gave to each of these aggregated principles.

• Accountability/liability: accountability refers to the idea that developers and deployers of AI technologies should be compliant with regulatory bodies. These actors should also be accountable for their actions and the impacts caused by their technologies.
• Beneficence/non-maleficence: beneficence and non-maleficence come from bioethics and medical ethics. In AI ethics, these principles state that human welfare (and harm aversion) should be the goal of AI-empowered technologies.
• Children and adolescents rights: this is the idea that we must protect the rights of children and adolescents. AI stakeholders should safeguard, respect, and be aware of the fragilities associated with young people.
• Dignity/human rights: this principle is based on the idea that all individuals deserve proper treatment and respect. In AI ethics, respect for human dignity and human rights (i.e., the Universal Declaration of Human Rights) are used (sometimes) interchangeably.
• Diversity/inclusion/pluralism/accessibility: this set of principles advocates the idea that the development and use of AI technologies should be done in an inclusive and accessible way, respecting the different ways that the human entity may come to express itself (gender, ethnicity, race, sexual orientation, disabilities, etc.).
• Freedom/autonomy/democratic values/technological sovereignty: this set of principles advocates the idea that the autonomy of human decision-making must be preserved during human-AI interactions, whether that choice is individual or the freedom to choose together, such as the inviolability of democratic rights and values, also being linked to technological self-sufficiency of nations/states.
• Human formation/education: such principles defend that human formation and education must be prioritized in our technological advances. AI technologies require considerable expertise to be produced and operated, and such knowledge should be accessible to all.
• Human-centeredness/alignment: such principles advocate that AI systems should be centered on and aligned with human values. This principle is also used as a "catch-all" category, many times being defined as a collection of "principles that are valued by humans" (e.g., freedom, privacy, non-discrimination, etc.).
• Intellectual property: this principle seeks to ground the property rights over AI products and their generated outputs.
• Justice/equity/fairness/non-discrimination: this set of principles upholds the idea of non-discrimination and bias mitigation (discriminatory algorithmic biases AI systems can be subject to). It defends that, regardless of the different sensitive attributes that may characterize an individual, algorithmic treatment should happen "fairly."
• Labor rights: labor rights are legal and human rights related to the labor relations between workers and employers. In AI ethics, this principle emphasizes that workers’ rights should be preserved regardless of whether labor relations are being mediated/augmented by AI technologies.
• Cooperation/fair competition/open source: this set of principles advocates different means by which joint actions can be established and cultivated between AI stakeholders to achieve common goals. It also relates to the free and open exchange of valuable AI assets (e.g., data, knowledge, patent rights, human resources).
• Privacy: the idea of privacy can be defined as the individual’s right to "expose oneself voluntarily, and to the extent desired, to the world." This principle is also related to data protection related-concepts such as data minimization, anonymity, informed consent, and others.
• Reliability/safety/security/trustworthiness: this set of principles upholds the idea that AI technologies should be reliable, in the sense that their use can be truly attested as safe and robust, promoting user trust and better acceptance of AI technologies.
• Sustainability: this principle can be interpreted as a manifestation of "intergenerational justice," wherein the welfare of future generations must be considered in AI development. In AI ethics, sustainability pertains to the notion that the advancement of AI technologies should be approached with an understanding of their enduring consequences, encompassing factors such as environmental impact and the preservation and well-being of non-human life.
• Transparency/explainability/auditability: this set of principles supports the idea that the use and development of AI technologies should be transparent for all interested stakeholders. Transparency can be related to "the transparency of an organization" or "the transparency of an algorithm." This set of principles is also related to the idea that such information should be understandable to nonexperts and, when necessary, subject to be audited.
• Truthfulness: this principle upholds the idea that AI technologies must provide truthful information. It is also related to the idea that people should not be deceived when interacting with AI systems.

These 17 principles contemplate all of the normative discourse we could interpret. At the end of this second phase, all documents received 13 features: origin country, world region, institution, institution type, year of publication, principles, principles definition, gender distribution, size, type I (nature/content), type II (form of regulation), type III (normative strength), type IV (impact scope), plus identifiers and attachments like document title, abstract, document URL, and related documents.

### Our tool

We used all information obtained during the second phase to create the dataset that feeds our visualization tool: an interactive dashboard. We created this dashboard using the Power BItool (https://www.airespucrs.org/en/worldwide-ai-ethics). We also developed a secondary dashboard (open source) using the Dash library (https://playground.airespucrs.org/worldwide-ai-ethics), an open source framework for building data visualization interfaces.

The main distinction between our tool and Hagendorff’s table^19^ and Fjeld et al.’s graphs^20^ lies in its interactivity and the flexibility to combine various filters without being confined to preconfigured orderings. This feature allows researchers to utilize the tool to examine and question specific characteristics present in their regions, to identify trends and behaviors, or to explore categories relevant to their research focus. It is worth noting that we were among the first to openly release our dataset, making our work accessible and reproducible.

Another distinguishing feature of our tool is its ability to condense large amounts of information into a single visualization panel. Our choice for such a way of presenting our data was to make it easier to interpret how certain features interact with others. While previous works demonstrate the statistical distribution of certain features, ours allows the user to see how these features vary and are interconnected.

### Limitations

As in past works, this analysis also suffers from a small sample. Our work represents a mere fraction of what our true global landscape on this matter is. Some of the main limitations we encountered during our work are the following.

(1) The limited scope of languages we were able to interpret represents a language bias, potentially excluding relevant perspectives.
(2) Publication bias is also a concern, as the focus on published guidelines may overlook valuable insights from ongoing discussions in other forms of media.
(3) The "guideline" scope excludes the academic work being done worldwide (i.e., we did not consider academic papers on AI ethics).
(4) The study’s temporal scope limits our understanding of past dynamics and trends in AI ethics that predate our window of analysis.
(5) Methodological limitations, such as data collection techniques and analysis frameworks, can influence the results and interpretations.
(6) The study may lack contextual information, failing to address the deeper social, cultural, and political aspects surrounding AI ethics discussions.
(7) Ethical considerations, particularly related to gender representation and bias, require further exploration. Our limited "male/female" analysis of gender distribution hides many problems related to gender inequality.

## Results and discussion

In the following sections, we pair our findings with the results of other studies to provide a dialogue with the literature.

### World regions and countries distribution

**Figure 2:**
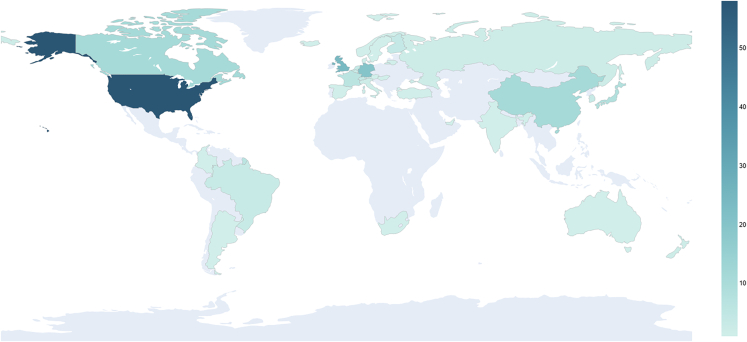
Number of published documents by country

Looking at the distribution among world regions (Figure 2) (aggregated by continent), we see that the bulk of produced documents come from Europe (especially countries from Western Europe, 63 = 31.5%, like the United Kingdom, 24 = 12%, and Germany, 20 = 10%), North America (United States of America, 58 = 29%, and Canada, 11 = 5.5%), which together represent a third of our sample size, and Asia (mostly represented by East Asian countries, 23 = 11.5%, like China, 11 = 5.5%, and Japan, 8 = 4%), while South America, Africa, and Oceania represent less than 4.5% of our sample, with countries like Brazil (3 = 1.5%) spearheading this portion of our distribution (Latin America, 7 = 3.5%). If it was not for the significant participation of intergovernmental organizations, like NATO, UN, and UNESCO, which represent 6% of our sample size (13 documents), other world regions/countries would be even more underrepresented. However, this still excludes states like the Holy See/Vatican City and Palestine.

When we examine our sample through a "country" level of granularity, we see that the bulk (13 countries = 77%) of our total sample size is represented by the United States of America, the United Kingdom (England, Scotland, Wales, and Northern Ireland have been considered as a country unit, even though technically this is not the case), Germany, Canada, China, Japan, France, Finland, Netherlands, Switzerland, Belgium, Brazil, and South Korea, while a myriad of 24 countries (12.5%) represents the remainder of our sample, along with intergovernmental organizations, like the EU (9 = 4.5%) and the UN (6 = 3%).

### Underrepresentation of world regions and countries distribution

We first would like to discuss this apparent unwavering distribution of documents into world regions/countries. Even with a sample size twice as large as the one analyzed by Jobin et al.,^3^ we seem unable to escape this result. However, we would like to defy this result by bringing other indicators.

For example, according to Savage,^26^ from 2016 to 2019: "China’s output of AI-related research increased by just over 120%, whereas output in the United States increased by almost 70%. In 2019 China published 102.161 AI-related papers, and the United States published 74.386." Also, based on our analysis of the AI Index 2022 Annual Report, the top three countries by the Vibrancy Ranking score were the United States, China, and India. This explains why almost a third of our sample size (58 documents) comes from the United States, but it does not account for the underrepresentation of countries like China (only 5.5% of our sample) and India (0.5%). Again, according to Zhang et al., China has far surpassed the United States in journal/conference publications and citations, while most of the "AI talent concentration" is found in India.

This underrepresentation, also found in previous studies,^3^^,^^19^ may be due to our language limitations, lack of representative databases, and unfamiliarity of how to find such documents.

Also, we argue that the "Guidelines for AI Technologies" scope hides much of the normative discourse done elsewhere. For example, the African continent is significantly underrepresented in our dataset (only one sample). However, according to Kiemde and Kora,^27^ 17 of the 55 African Union member states possess data protection and privacy legislation, while Mauritius has announced the establishment of a National AI Council, also being the first African state to present an AI strategy/roadmap. Kiemde and Kora also demonstrate in their review a collection of published papers and documents about AI ethics in Africa and other underrepresented countries,^28^^,^^29^^,^^30^^,^^31^^,^^32^ which helps us to show that this type of discourse is present in the African states and probably in all other places that did not show up in our sample. They only do not come in the format we were first looking for or were not caught by our method.

### Institutional distribution

**Figure 3:**
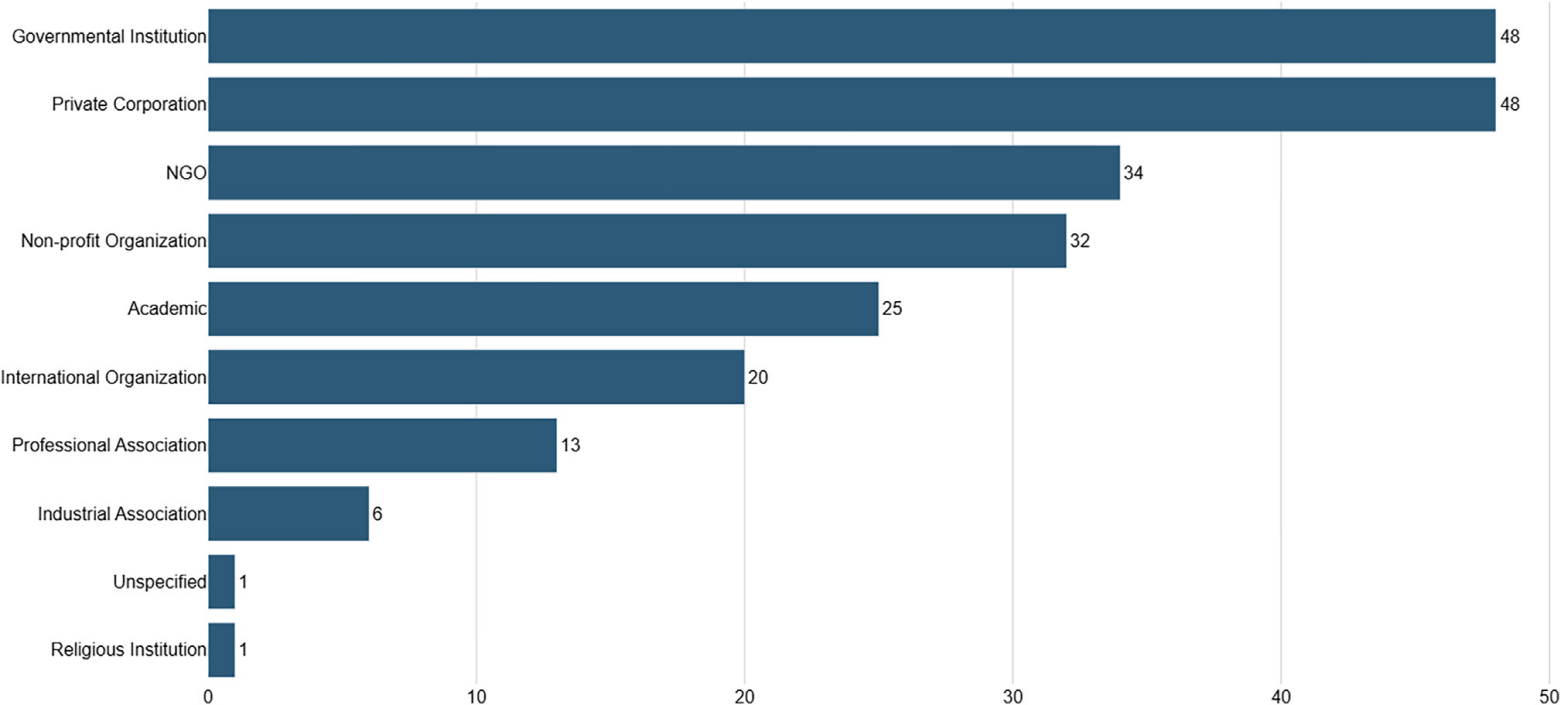
Publications by institution types

Switching our gaze to institution types (Figure 3), except for institutions like IBM (5), Microsoft (4), and UNESCO (3), most other institutions do not have more than two published documents. We can also see that the bulk of our sample was produced by governmental institutions and private corporations (48%), followed by CSOs/NGOs (17%), non-profit organizations (16%), and academic institutions (12.5%). However, this trend only follows if we look at the totality of our sample size. If we look at documents produced by continents, for example, in North America (69), private corporations (24 = 34.7%) and non-profit organizations (18 = 26%) produced most documents, followed by governmental institutions (12 = 17.4%). Meanwhile, when we look at Europe, the global trend is restored.

An in-depth analysis segmented by countries shows that the engagement of particular AI stakeholders (i.e., institution types) differs between countries. For example, in China (11), the majority of documents are produced by academic institutions (5 = 45.4%), while in Germany (20), most samples came from private corporations (6 = 30%) and CSOs/NGOs (4 = 20%). Also, the only document produced by a religious institution in our sample is the "Rome Call For AI Ethics," produced by the Pontifical Academy for Life (Vatican City).

### The hegemony of state and private sectors

Our results mirror the findings of Jobin et al.^3^ and Fjeld et al.,^20^ where most of our sample comes from private institutions (24%) and governmental organizations (24%).

This equal presence of both state and private stakeholders in the current normative discourse may be related to the expanse and success of the tech industry, as already stated in our introduction (>$90 billion USD invested in 2021).^2^ Meanwhile, nowadays, most AI breakthroughs come from the industry.^33^^,^^34^^,^^35^ This is an industry that, seeing the demands for regulation and accountability from civil society, quickly reacted by proposing the rules that should (allegedly) guide their progress. Many of such promises are, perhaps, genuine. However, when governments and private institutions have "the same weight" in our sample, attention to the matter seems needed, especially when many of these technologies remain in gray areas of regulation.

#### Gender distribution

When we examined gender distribution among authors, first, we noticed that 66% (132) of our samples have no authorship information. Second, we saw that the distribution of authors with "male" names was favorable in the remaining portion of our dataset (549 = 66% male, 281 = 34% female). While academic institutions (62% male, 38% female) and non-profit organizations (65% male, 34% female) are the less disparate institutions, they still fall short of the 1:1 parity ratio.

### Gender disparity in authorship

Gender differences between male and female authors in our sample, as previously noted by Hagendorff,^19^ are significant. However, it is worth highlighting that the number of unidentified authors may still obscure a more pronounced inequality.

Regarding our methods of inference, it is important to acknowledge that gender prediction methods still exhibit a gender bias error rate^36^ that we were unable to address. While name-based analyses are generally considered sound practices,^37^^,^^38^ they are limited in capturing non-binary gender accounts and fail to cover cases of self-declaration (e.g., non-binary, gender-fluid, queer, or transgender).

Also, we would like to point out in the writing of this document, it is hard to find present-day data about gender disparity in the workplace and academia since many organizations do not provide this information. However, we would like to mention some external sources that help support our findings as a reflection of the world. For example, in the AI Now report of 2018, Whittaker et al.^17^ showed that 80% of the professors at the world’s leading universities are male. Also, according to the US National Center for Education Statistics, between 2008 and 2017, women earned only 32% of undergraduate degrees in STEM (even given that more women graduate in the United States than men, 60% more) and 18% of degrees in computer science. In the United Kingdom, women account for only 16% of the tech industry, and in Silicon Valley, the male/female proportion is 4:1.

Lastly, Google is one of the few big tech companies that make their internal demographic distribution publicly available.^39^ In their 2022 report, Google proudly stated that "Black+ representation grown 2x faster than Googlers overall"; however, men (62.5%) are still more hired than women (37.5%) at Google (globally), and leadership positions are still predominantly held by men (69.4%), while Black, Latin, and Native American women represent only 19.2% of their female workforce.

### Year of publication distribution

**Figure 4:**
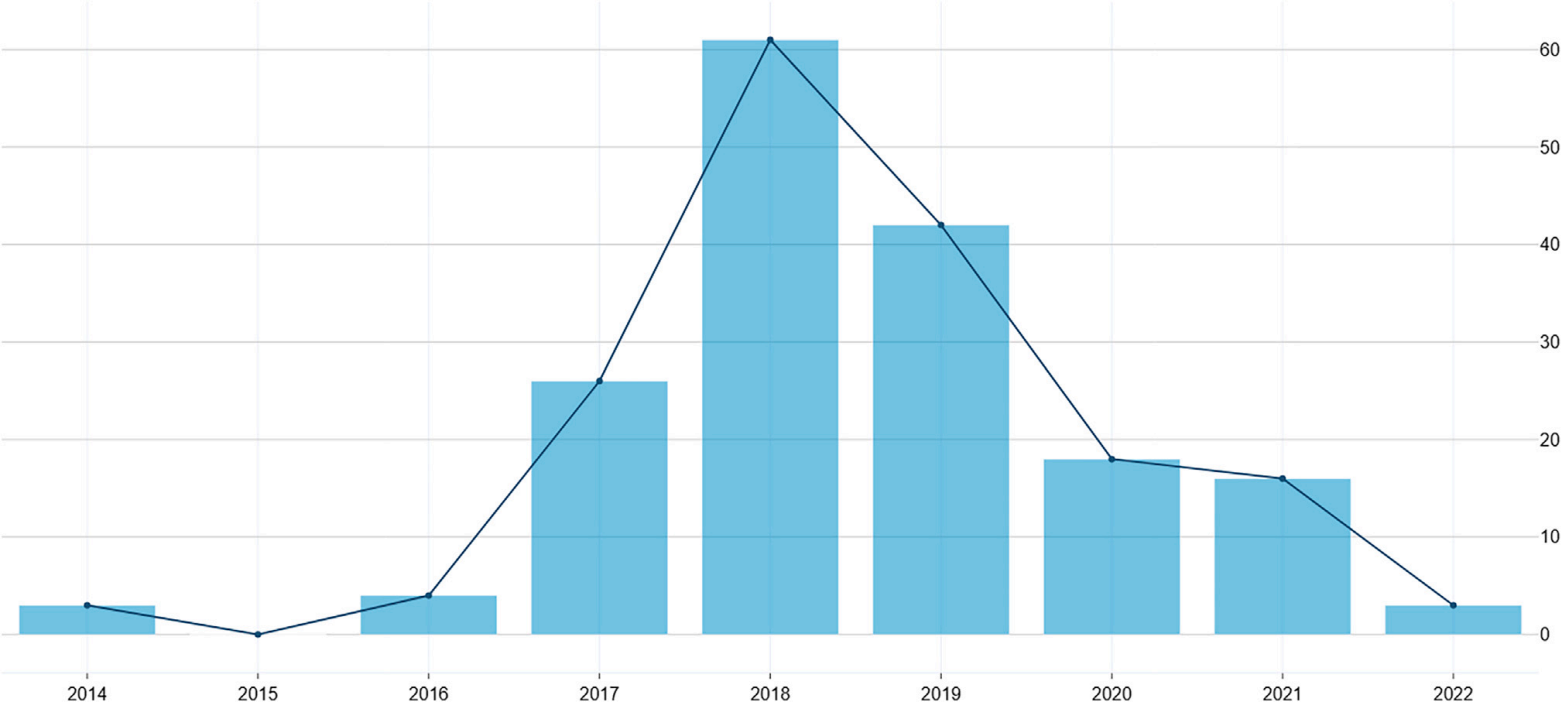
Number of publications per year

Concerning the year of publication of the documents from our sample, one can see that the majority of them (129 = 64.5%) were published between 2017 and 2019. What we call the "AI ethics boom" constitutes the significant production of documents in the year 2018, which represents 30.5% (61) of our entire sample (Figure 4).

#### The AI ethics boom, shifts in attention, and historical marks

The fact that almost a third of our sample (30.5%) got published in 2018 (64.5% if extended from 2017 to 2019) is worth contextualizing. The AI Index report also points to this trend, where since 2014, we had a five-time increase in publications related to AI ethics, where topics like algorithmic fairness have stopped being only academic objects of research and actual AI industry areas of R&D.^2^

It is also interesting to see the shift of interest during the timeline we analyzed. In 2014, the top-cited principles were fairness, reliability, and dignity (transparency was not even in the top 10 at this time), and in 2016, accountability, beneficence, and privacy received more attention (accountability being the number one concern of documents published in 2017). But in 2018, transparency (explainable AI/XAI, mechanistic interpretability) became the dominant topic of concern.

### What factors could explain this shift in attention? We can start by analyzing past events that may be relevant to the field

For example, some high-profile cases worth mentioning are the COMPAS software use, which in the year 2016, Angwin et al.^40^ showed that "blacks are almost twice as likely as whites to be labeled a higher risk but not re-offend." Also, in 2018, we had the first case of a human killed by an Uber self-driving car.^41^ In the same year, the Cambridge Analytica case gained considerable media attention, where the use of personal data (without consent) allowed for personal profiling and targeted political advertising during elections.^42^^,^^43^ We can also mention relevant works that helped cement the AI ethics field as a popular area of research, like the book *Weapons of Math Destruction*.^44^

All these events and many others may have helped bring this burst of interest to the field. Perhaps some of these events could come to explain the swings of attention on AI ethics. And of course, this increase in popularity may also be related to the increased funding that AI research (where AI ethics remains as a sub-field) received in the last decade.^2^

### Typological categories

Regarding the previously defined typological categories, when looking at the document’s nature/content, we found that the majority of our sample is from the normative type (96%), which a third of the time also presents descriptive contents (55.5%) and, more rarely, practical implementations (2%).

When we look at the form of regulation proposed by the documents of our sample, more than half (56%) are only recommendations to different AI stakeholders, while 24% possess self-regulatory/voluntary self-commitment style guidelines, and only 20% propose a form of regulation administered by a given state/country.

This lack of convergence to a more "government-based" form of regulation reflects in the normative strength of these documents, where the vast majority (98%) only serve as "soft laws," i.e., guidelines that do not entail any form of a legal obligation, while only 4.5% propose stricter regulation. Since only governmental institutions can create legally binding norms (other institutions lack this power), and they produced only 24% of our sample, some may argue that this imbalance lies in this fact. However, by filtering only the documents produced by governmental institutions, the disproportion does not go away, with only 18.7% of samples proposing legally binding forms of regulation. The countries on the front of this still weak trend are Canada, Germany, and the United Kingdom, with Australia, Norway, and the United States coming right behind.

Our last typology group is impact scope. Looking at the totality of our sample size, we see that short-term (47%) and "mid-term" (i.e., short-term and long-term = 52%) prevail over more long-term preoccupations (2%). When we filter our sample by impact scope and institution type, it seems to us that private corporations think more about the short-term (33%), governmental institutions about the short/long-term (28%), and academic (66%) and non-profit organizations (33%) with the long-term impacts of AI technologies.

### Definitions, lack of tools, the legislative push, and uncertain risks

In regard to the nature/content of our samples, we see that only 55.5% of documents (111) seek to define what is the object of their discourse, i.e., "we are talking about autonomous intelligent systems, and this is what we understand as an autonomous intelligent system." This is a curious phenomenon, more so if we acknowledge that there is no consensual definition of what "artificial intelligence" is and what it is not.^45^ There are many interpretations and contesting definitions, which may prove to be a challenge for regulating organizations. For example, if you choose to define AI as only "systems that can learn," you will leave outside your scope of regulation an entire family of systems that do not learn (rule-based systems) but can still act "intelligently" and autonomously.

Meanwhile, as already stated by Fjeld et al.,^20^ there is a gap between established principles and their actual application. In our sample, most of the documents only prescribe normative claims without the means to achieve them, while the effectiveness of more practical methodologies, in the majority of cases, remains extra empirical. With this, we see a field with a significant lack of practical implementations^46^ that could support its normative claims.

This fact may become more alarming when we look at the distribution of government documents that opt for "soft" forms of regulation (91.6%). The critique that "ethical principles are not enough to govern the AI industry" is not a new one.^3^^,^^19^^,^^47^^,^^48^^,^^49^ However, perhaps those critiques have not yet permeated the mainstream community, which, by our analysis, is still largely based on principles detached from observable metrics or practical implementations.

However, even if most countries in our sample seem to opt for legally non-binding forms of regulation, there seems to be a growing adoption/proposition of stricter solutions. The idea that "ethics" and "compliance" are separate domains seems to get ever-growing acknowledgment by countries such as Canada, Germany, and the United Kingdom (which comprise 66.6% of our total "legally binding" sample), while according to Zhang et al., the legislative records on AI-related bills grew from just one in 2016 to 18 in 2021, with Spain, the United Kingdom, and the United States being the top three "proto-AI-legislators" from 2021, showing that in fact, we may be passing into a transitioning phase where these principles may soon be transformed into actual laws.

We would also like to point out the seemingly low attention given to the long-term impacts of AI (1.5%). Even though there is a considerable amount of work produced on the matter,^50^^,^^51^^,^^52^^,^^53^^,^^54^^,^^55^ many times the terms "safety," "alignment," or "human-level AI" are generically dismissed as not serious or as Stuart Russell^56^ would say, "myths and moonshine." Possible explanations for this fact could be the following: (1) the AI community does not find these problems real; (2) the AI community does not find these problems urgent; (3) the AI community thinks we have more urgent problems at hand; or even (4) that the AI community does not know about such issues. Regardless of their urgency, we argue that the current lack of attention given to safety-related topics in the field is alarming. For example, if we look at the distribution of papers submitted in the NeurIPS 2021, approximately 2% were safety related (e.g., AI safety, ML fairness, privacy, interpretability).

### Principle distribution

**Figure 5:**
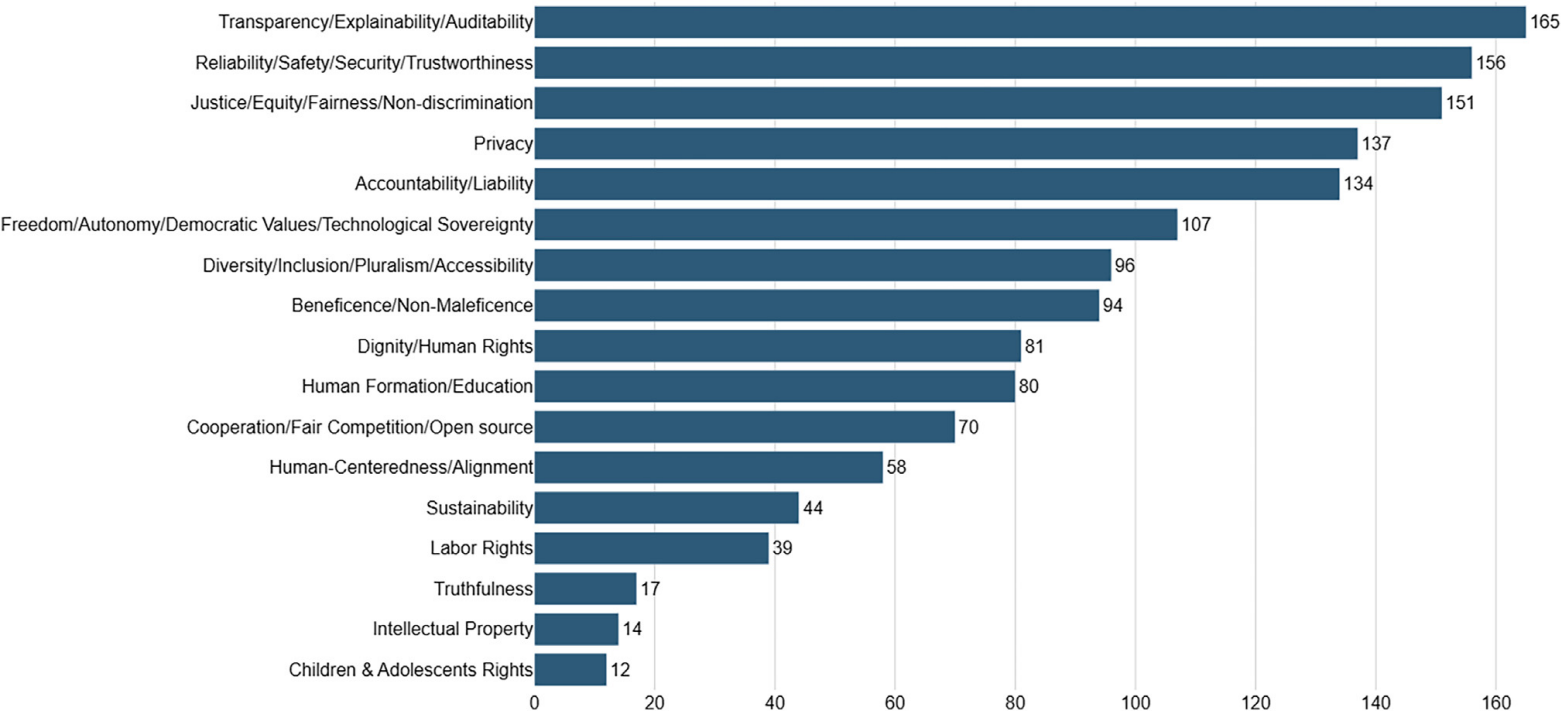
Number of times an aggregated principle was cited

Examining the distribution of principles among our total sample size, we arrive at the following results: the top five principles advocated in the documents of our sample are similar to the results shown by Jobin et al.^3^ and Hagendorff,^19^ with the addition of reliability/safety/security/trustworthiness (78%), which also was top five in Fjeld et al.’s^20^ meta-analysis (80%) (Figure 5).

Looking at principle distribution filtered by continent, the top five principles remain the same in both North America and Europe, but the Asian continent introduces the principle of beneficence/non-maleficence as its fifth (74%) most cited principle, putting accountability/liability in sixth place (70%). Filtering our results by country, we see no change in the top five principles when comparing USA and the United Kingdom. However, looking under the top five principles, we begin to see differences, like freedom/autonomy/democratic values/technological sovereignty (38%) and beneficence/non-maleficence (34.4%) being the sixth and seventh most cited principles in the EUA, and cooperation/fair competition/open source (45.8%) and diversity/inclusion/pluralism/accessibility (41.6%) being sixth and seventh most cited principles in the UK.

When examining principle distribution filtered by institution type, we also can find many insights. For example, looking at our total sample, we notice that the main preoccupation of governmental institutions (worldwide) is the need for transparent systems (89.5%), private corporations mainly advocate for reliability (87.5%), and CSOs/NGOs primarily defend the principle of fairness (88.2%).

### Hidden costs and divergences

When looking at some of our least mentioned principles, like labor rights, sustainability, and truthfulness, we reaffirm the problems stated by Hagendorff,^19^ where the lack of attention given to questions related to the costs and misuse of our current AI technologies is overlooked in much of these guidelines’ discourse.

For example, modern AI systems have the potential to incur massive energy consumption during their development, depending on the availability of large amounts of computational resources and energy. While the carbon footprint required to fuel these processes is known to be large,^57^^,^^58^^,^^59^ little is known about the costs beyond the CO_2_ metric (e.g., the depletion of natural resources related to the construction of hardware^60^^,^^61^).

Meanwhile, while many point to the possibility of massive labor displacement due to innovation,^62^^,^^63^^,^^64^ proposed measures to avoid mass unemployment/monopolies are infantile at best.^65^ Simultaneously, as the field of generative AI rapidly grows, the not-so-talked-about principle of truthfulness gains a renewed sense of urgency, which makes the discourse on much of these guidelines outdated.

Another point we would like to bring attention to, as done by Jobin et al.^3^ and Fjeld et al.,^20^ is the divergence concerning how these principles are defined. Our tools bring all definitions given by each document to the mentioned principles, which allows for a more diverse comparison of how these abstract objects are presented. Here, we bring some cases that most sparked curiosity, reminding us that this analysis is partial to our subjective interpretation of how the discourse surrounding these principles varies. The reader may well find other more intriguing discrepancies by searching our tool.

For example, when examining transparency/explainability/auditability, the definition proposed in *ARCC*: *An Ethical Framework for Artificial Intelligence*^66^ states the following:

Promote algorithmic transparency and algorithmic audit, to achieve understandable and explainable AI systems. Explain the decisions assisted/made by AI systems when appropriate. Ensure individuals’ right to know, and provide users with sufficient information concerning the AI system’s purpose, function, limitation, and impact.

While the one provided by *A Practical Guide to Responsible Artificial Intelligence (AI)*^67^ says the following (about the same aggregated principle):

To instill trust in AI systems, people must be enabled to look under the hood at their underlying models, explore the data used to train them, expose the reasoning behind each decision, and provide coherent explanations to all stakeholders promptly. These explanations should be tailored to the different stakeholders, including regulators, data scientists, business sponsors, and end consumers.

If we take a look at human-centeredness/alignment, in *Data*, *Responsibly (Vol*. *1) Mirror*, *Mirror*,^68^ we find the following recommendation: “Maybe what we need instead is to ground the design of AI systems in people. Using the data of the people, collected and deployed with an equitable methodology as determined by the people, to create technology that is beneficial for the people."

While in "*Everyday Ethics for Artificial Intelligence*,"^69^ the following norm is suggested: "AI should be designed to align with the norms and values of your user group in mind."

Other examples we can mention are as follows.

• *Tieto’s AI Ethics Guidelines*^70^ takes a different take on explainability, saying its systems "can be explained and explain itself," making it a stakeholder in the accountability chain.
• The “The Toronto Declaration”^7^ gives an extensive and non-exhaustive definition of what discrimination means under international laws, while most other documents resume themselves by only citing the concept, leaving the concept open to interpretation.
• In "Artificial Intelligence and Machine Learning: Policy Paper,"^15^ fairness is related to the idea of "AI provides socio-economic opportunities for all" (benefits), while in *Trustworthy AI in Aotearoa*: *AI Principles*,^71^ fairness is defined as "AI systems do not unjustly harm" (impacts).
• While some documents (e.g., "Telefónica's Approach to the Responsible Use of AI")^11^ state how privacy and security are essential for AI systems developments, only a few define (e.g., "Big Data, Artificial Intelligence, Machine Learning, and Data Protection")^72^ what "good privacy criteria" are.
• While most documents interpret accountability/liability as "developers being responsible for their projects" (e.g., "Declaration of Ethical Principles for AI in Latin America"),^73^ some documents also put this responsibility on users and even algorithms (e.g., "The Ethics of Code: Developing AI for Business with Five Core Principles"^74^).

### Final remarks

In this work, we sought to bring new data, insights, tools, typologies, and our evaluation of the current state of AI ethics guidelines as an open access database to the community. We also sought to substantiate our analysis with past works, presenting the next step in meta-analytical studies related to this field. To do so, we collected data from documents throughout the world. Although still lacking in diversity, we argue that these 200 documents paint the picture of a world needing clear and enforceable rules for AI development. It is also the picture of a world where many voices are still missing.

From these analyses, it was possible to diagnose at least 17 groups of principles listed among the 200 guidelines analyzed. This information certainly contributes as a guide for the discussions that are taking place on how to regulate artificial intelligence, indicating what objectives/minimum requirements should be protected by future legislation. Besides, by making our work and results open, other researchers can easily extend and replicate our work. Therefore, we hope our tool and results can help progress the refinement and creation of new normative tools for governing AI technologies.

## Experimental procedures

### Resource availability

As primary sources for our sample, we utilized two public repositories: AlgorithmWatch’s "AI Ethics Guidelines Global Inventory" and the "Linking Artificial Intelligence Principles" (LAIP) guidelines. The AlgorithmWatch repository encompassed 167 documents (https://inventory.algorithmwatch.org/), whereas the LAIP repository had 90 (https://www.linking-ai-principles.org/).

To create our dataset, we conducted a thorough examination of duplicate samples across the two repositories. Once duplicates were identified and removed, we proceeded to augment our collection of documents by employing web search engines and web scraping techniques. We used search strings such as "Artificial Intelligence Principles" and "Artificial Intelligence Guidelines" in this process. We specifically focused on gathering documents written or translated into English, Portuguese, French, German, and Spanish.

We defined the documents used as "Guidelines for AI Technologies." By this definition, we encompassed ethical guidelines, recommendations, policy frameworks, legal landmarks, codes of conduct, practical guides, and other documents related to the use and development of AI technologies. We also considered the various subfields and applications related to AI technologies, including data science, machine learning, robotics, and software development.

We carried out our analysis in two phases. In the first phase, our team read, translated, and extracted pre-defined features from these documents. These features encompassed characteristics such as the responsible institution, the geographical location of the institution (country/world region), the type of institution (academic, non-profit, governmental, etc.), the publication year, the advocated principles within the document, the gender distribution among authors, and the document size. We inferred genders using a name + nationality approach. We inferred the most likely nationality of names using the Nationalize.io API (https://nationalize.io/), while for gender we used the Genderize.io API (https://genderize.io/).

In the second phase, one team member reviewed the entire sample to refine the list of principles and created typologies. We identified 17 different principles after aggregating resonating principles via an n-gram analysis. Every document received 13 features: origin country, world region, institution, institution type, year of publication, principles, principles definition, gender distribution, size, nature/content (descriptive, normative, or practical), form of regulation (government-regulation, self-regulation/voluntary self-commitment, or recommendation), normative strength (legally non-binding guidelines or legally binding horizontal regulations), impact scope (short-termism, long-termism, or both), plus identifiers and attachments like document title, abstract, document URL, and related documents.

#### Lead contact

The lead contact is the author Nicholas Kluge Corrêa, from the Graduate Program in Philosophy at the Pontifical Catholic University of Rio Grande do Sul/University of Bonn, Porto Alegre/Bonn, Rio Grande do Sul/North Rhine-Westphalia, Brazil/Germany. His e-mail is nicholas@airespucrs.org.

#### Materials availability

We used the created dataset to develop a visualization tool using Power BI (https://www.airespucrs.org/worldwide-ai-ethics) and the Dash library (https://playground.airespucrs.org/worldwide-ai-ethics). The developed dashboard allows researchers to explore and analyze the data by combining filters that update the visualization to their selected focus.

#### Data and code availability

The reader can find our code implementation on GitHub (https://github.com/Nkluge-correa/worldwide_AI-ethics). Hence, the dataset and source code (Dash implementation) are openly available, allowing reproducible and extendable results. Also, the original data have been deposited at Zenodo (https://zenodo.org/record/8172350) https://doi.org/10.5281/zenodo.8172350.
